# Participation of the adenosine salvage pathway and cyclic AMP modulation in oocyte energy metabolism

**DOI:** 10.1038/s41598-019-54693-y

**Published:** 2019-12-05

**Authors:** Dulama Richani, Cathy F. Lavea, Raji Kanakkaparambil, Angelique H. Riepsamen, Michael J. Bertoldo, Sonia Bustamante, Robert B. Gilchrist

**Affiliations:** 1 School of Women’s and Children’s Health, Fertility & Research Centre, University of New South Wales Sydney, Sydney, Australia; 2 Department of Veterinary Physiology, Kerala Veterinary and Animal Sciences University, Kerala, India; 3 School of Medical Sciences, University of New South Wales Sydney, Sydney, Australia; 4 Bioanalytical Mass Spectrometry Facility, University of New South Wales Sydney, Sydney, Australia

**Keywords:** Cell biology, Biochemistry

## Abstract

A follicular spike in cyclic AMP (cAMP) and its subsequent degradation to AMP promotes oocyte maturation and ovulation. *In vitro* matured (IVM) oocytes do not receive the cAMP increase that occurs *in vivo*, and artificial elevation of cAMP in IVM cumulus-oocyte complexes improves oocyte developmental potential. This study examined whether mouse oocytes can use the cAMP degradation product AMP to generate ATP via the adenosine salvage pathway, and examined whether pharmacological elevation of cAMP in IVM cumulus-oocyte complexes alters ATP levels. Oocytes cultured with isotopic ^13^C_5_-AMP dose-dependently produced ^13^C_5_-ATP, however total cellular ATP remained constant. Pharmacological elevation of cAMP using forskolin and IBMX prior to IVM decreased oocyte ATP and ATP:ADP ratio, and promoted activity of the energy regulator AMPK. Conversely, cumulus cells exhibited higher ATP and no change in AMPK. Culture of oocytes without their cumulus cells or inhibition of their gap-junctional communication yielded lower oocyte ^13^C_5_-ATP, indicating that cumulus cells facilitate ATP production via the adenosine salvage pathway. In conclusion, this study demonstrates that mouse oocytes can generate ATP from AMP via the adenosine salvage pathway, and cAMP elevation alters adenine nucleotide metabolism and may provide AMP for energy production via the adenosine salvage pathway during the energetically demanding process of meiotic maturation.

## Introduction

Within the ovarian antral follicle, the oocyte is surrounded by cumulus cells that are connected to the oocyte and each other by gap junctions, forming an entity called the cumulus-oocyte complex (COC)^1^. Cyclic adenosine monophosphate (cAMP) is a key regulator of mammalian oocyte maturation. It plays paradoxical roles within the oocyte and cumulus cells to orchestrate oocyte meiotic arrest and resumption^2^. The oocyte synthesises cAMP through constitutive activation of a G-protein coupled receptor, which acts to maintain meiotic arrest by supressing activity of the maturation-promoting factor^2^, and in addition cumulus cells likely supply the oocyte with cAMP^3^. Cyclic AMP is acutely and transiently upregulated in mural granulosa cells and in the oocyte in response to the ovulatory luteinising hormone (LH) surge^4,5^, initiating signalling events that promote oocyte meiotic resumption. These include (i) upregulation of the EGF-like peptide signalling cascade in granulosa and cumulus cells^6^, and (ii) downregulation of follicular cGMP levels^7,8^. Unlike the transient spike in COC cAMP that naturally occurs in response to the preovulatory LH surge, COCs matured *in vitro* exhibit a precipitous decline in cAMP^9^, thus the intricate molecular signals which are normally upregulated in cumulus cells in response to cAMP (e.g. EGF-like peptides) to orchestrate oocyte maturation are deficient.

**Figure 1:**
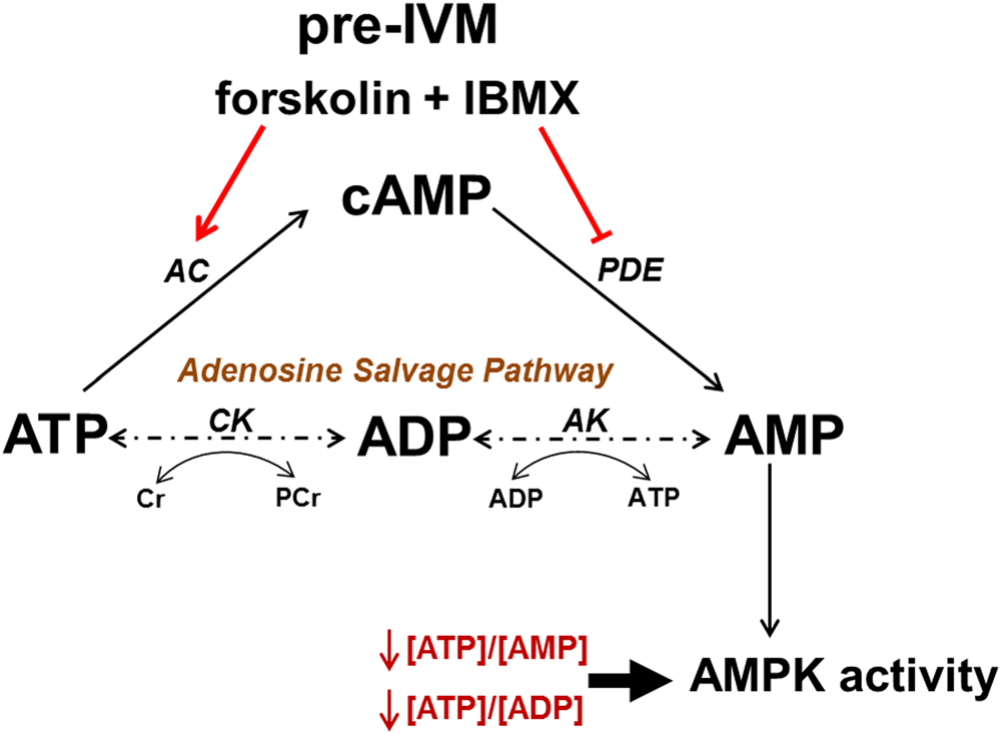
Cellular adenosine metabolism in relation to cAMP-elevating pre-IVM treatment. COC cAMP increases during the peri-ovular period and through pharmacological elevation during pre-IVM. Cyclic AMP is generated by adenylate cyclase (AC) from its substrate ATP and is hydrolysed to AMP by phosphodiesterases (PDE). AMP can be recycled to ATP via the adenosine salvage pathway. The energy sensing enzyme AMP-activated protein kinase (AMPK) is activated by shifts in ATP:AMP and ATP:ADP ratios. CK, creatine kinase; AK, adenylate kinase; Cr, creatine; PCr, phosphocreatine; IBMX, 3-isobutyl-1-methylxanthine; IVM, oocyte *in vitro* maturation.

In many species, oocytes can be matured *in vitro* via the reproductive technology *in vitro* maturation (IVM), whereby immature germinal vesicle stage COCs are collected with little to no hormonal stimulation and cultured until the oocyte reaches metaphase II^10,11^. IVM is clinically attractive but has a lower efficiency at generating pregnancies relative to conventional IVF^12^. The artificial upregulation of cAMP in IVM COCs has shown potential to improving pregnancy rates, although further refinement on such approaches are warranted^13^. One of the more established approaches to cAMP modulation of IVM COCs involves the incorporation of a pre-maturation phase, usually termed “pre-IVM”, prior to IVM whereby COCs are treated during pre-IVM with exogenous cAMP or cAMP modulating agents that cause a large spike in cellular cAMP^14,15^. We developed a cAMP-modulated IVM system that incorporates a short pre-IVM phase, wherein COCs are treated with the pharmacological cAMP modulators 3-isobutyl-1-methylxanthine (IBMX) and forskolin^9,16^. Forskolin (FSK) is a potent stimulator of cAMP synthesis that acts by activating adenylate cyclase, an enzyme that catalyses the conversion of ATP to cAMP^17^. IBMX is a broad spectrum inhibitor of cyclic nucleotide phosphodiesterases, the enzymes that hydrolyse cAMP to AMP^18^ (Fig. 1). This FSK/IBMX pre-IVM phase has been shown to increase COC cAMP levels substantially, thus mimicking to some extent the *in vivo* spike in cAMP caused by the gonadotrophin surge^9,19^. Several studies have shown FSK/IBMX pre-IVM can significantly improve oocyte quality as it leads to enhanced subsequent blastocyst development, blastocyst quality and pregnancy rates, relative to standard IVM (i.e. lacking pre-IVM)^9,16,20–22^, suggesting that this may be one approach to bridge the efficiency gap between IVM and IVF and therefore having clinical and commercial relevance.

The metabolic consequences of cAMP modulation in the COC remain poorly understood. As illustrated (Fig. 1), FSK/IBMX treatment promotes the consumption and generation of adenine nucleotides. ATP is used as a substrate for cAMP synthesis by forskolin, hence FSK/IBMX pre-IVM would be expected to lead to depletion of oocyte ATP. Degradation of cAMP is required for meiotic resumption to occur^2^, and its degradation product is 5′-AMP. A recent study has demonstrated that bovine oocytes may have the capacity to utilise AMP for ATP production via the adenosine salvage pathway^23^, a two-step enzymatic process in which AMP can be phosphorylated to ADP by adenylate kinase, and ADP is phosphorylated to ATP by creatine kinase (Fig. 1). Hence, natural or pharmacological elevation of COC cAMP may enable the oocyte to utilise AMP for energy production, particularly at a time of ATP reduction. AMP, ADP and ATP are also modulators of AMP-activated protein kinase (AMPK) which is an energy sensing enzyme that directs metabolic changes in response to cellular energy status by controlling the activity of key rate-limiting enzymes involved in lipid and carbohydrate metabolism^24^. AMPK activity is allosterically regulated by the adenine nucleotides AMP, ADP, and ATP which compete for binding on AMPK’s gamma subunit. Binding of ATP to the gamma subunit elicits structural changes allowing phosphatases to access Thr-172, while binding of AMP or ADP elicit conformational changes allowing greater affinity to AMPK’s upstream kinase LKB1, and therefore greater activity induced by post-translational modification^25^. Hence, alterations in cellular [ATP:ADP] or [ATP:AMP] ratios significantly influence AMPK activity. Despite the prominent role of AMPK in regulating cellular energy metabolism, investigation of the impact of COC cAMP modulation on AMPK activity is lacking.

This study aimed to investigate whether the adenosine salvage pathway is active in the mouse oocyte, and whether upregulation of cAMP in the COC impacts ATP production.

## Results

### The oocyte produces ATP from AMP via the adenosine salvage pathway

**Figure 2:**
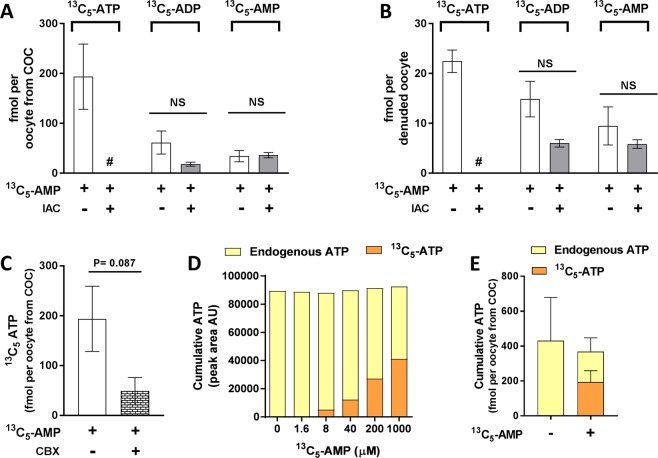
Oocytes utilise AMP for ATP production via the adenosine salvage pathway. Iodoacetamide (IAC) was used to inhibit creatine kinase activity in order to perturb the interconversion of ADP and ATP. COCs (**A**) or denuded oocytes (**B**) were cultured for 3 h ± ^13^C_5_-AMP and then intra-oocyte ^13^C_5_-AMP, ^13^C_5_-ADP and ^13^C_5_-ATP were measured by LC-MS/MS (n = 4 biological replicates). COCs were cultured with ^13^C_5_-AMP ± the gap-junction uncoupler carbenoxolone (CBX) for 3 h and intra-oocyte ^13^C_5_-ATP was measured (n = 4 biological replicates) (**C**). COCs were cultured for 3 h with increasing concentrations of ^13^C_5_-AMP and the peak areas of intra-oocyte ^13^C_5_-ATP and endogenous ATP were measured (n = 1 biological replicate) (**D**). COCs were cultured for 3 h ± ^13^C_5_-AMP (1 mM) and intra-oocyte ^13^C_5_-ATP was measured (n = 4 biological replicates) (**E**). ns, not significantly different (P ˃ 0.05, t-test), ^#^Not detected; data are mean ± SEM.

The ability of the oocyte to use AMP for ATP generation via the adenosine salvage pathway was examined by culturing COCs or DOs with 1 mM isotopic-labelled AMP (^13^C_5_-AMP), and measuring its conversion to ^13^C_5_-ADP and ^13^C_5_-ATP. In oocytes cultured either with their cumulus cells intact (oocytes from COCs) or as denuded oocytes, uptake of ^13^C_5_-AMP and its conversion to ^13^C_5_-ADP and ^13^C_5_-ATP was observed (Fig. 2A,B). Intact COCs were notably more efficient at generating ATP in the oocyte than oocytes denuded of cumulus cells. Treatment of COCs with ^13^C_5_-AMP resulted in ~8-fold more intra-oocyte ^13^C_5_-ATP than treatment of DOs (Fig. 2A,B). In addition, in the case of COCs, the proportion of intra-oocyte ^13^C_5_-ATP relative to the total amount of isotopic-labelled nucleotides was 67%. By contrast in denuded oocytes, ^13^C_5_-ATP makes up only 48% of the total isotopic nucleotides. Blockage of cumulus-oocyte gap-junctional communication using CBX led to a 3.8-fold reduction in generation of ATP from AMP, however this was not statistically significant (p = 0.087) (Fig. 2C).

Iodoacetamide (IAC) was used to inhibit the adenosine salvage pathway by inhibiting creatine kinase activity and thus the inter-conversion of ADP and ATP. Exposure to IAC did not affect ^13^C_5_-AMP uptake by COCs or DOs, or its conversion to ^13^C_5_-ADP, however its conversion to ^13^C_5_-ATP was ablated in both oocytes from COCs and in DOs (Fig. 2A,B). Accumulation of ADP was not seen in the presence of IAC, perhaps suggesting that nucleotide homeostasis may have been maintained by an equilibrium shift favouring the conversion of AMP to either adenosine or inosine monophosphate in the purine nucleotide biosynthetic pathway^26^. To assess the effect of increasing the availability of AMP on oocyte ATP levels (Fig. 2D,E), COCs were cultured with increasing doses of ^13^C_5_-AMP. A dose-dependent increase in intra-oocyte ^13^C_5_-ATP peak area, with a corresponding decrease in endogenous ATP peak area was observed, such that the net amount of ATP detected was constant (Fig. 2D). Cumulative intra-oocyte ATP was quantified following culture ± the top dose of 1 mM ^13^C_5_-AMP. The same trend was seen whereby the uptake and conversion of ^13^C_5_-AMP to ^13^C_5_-ATP led to a decrease in endogenous ATP which resulted in a cumulative ATP level (^13^C_5_-AMP + endogenous ATP) similar to endogenous ATP in oocytes not given ^13^C_5_-AMP (Fig. 2E).

### Elevated COC cAMP depletes intra-oocyte ATP and alters AMPK protein

**Figure 3:**
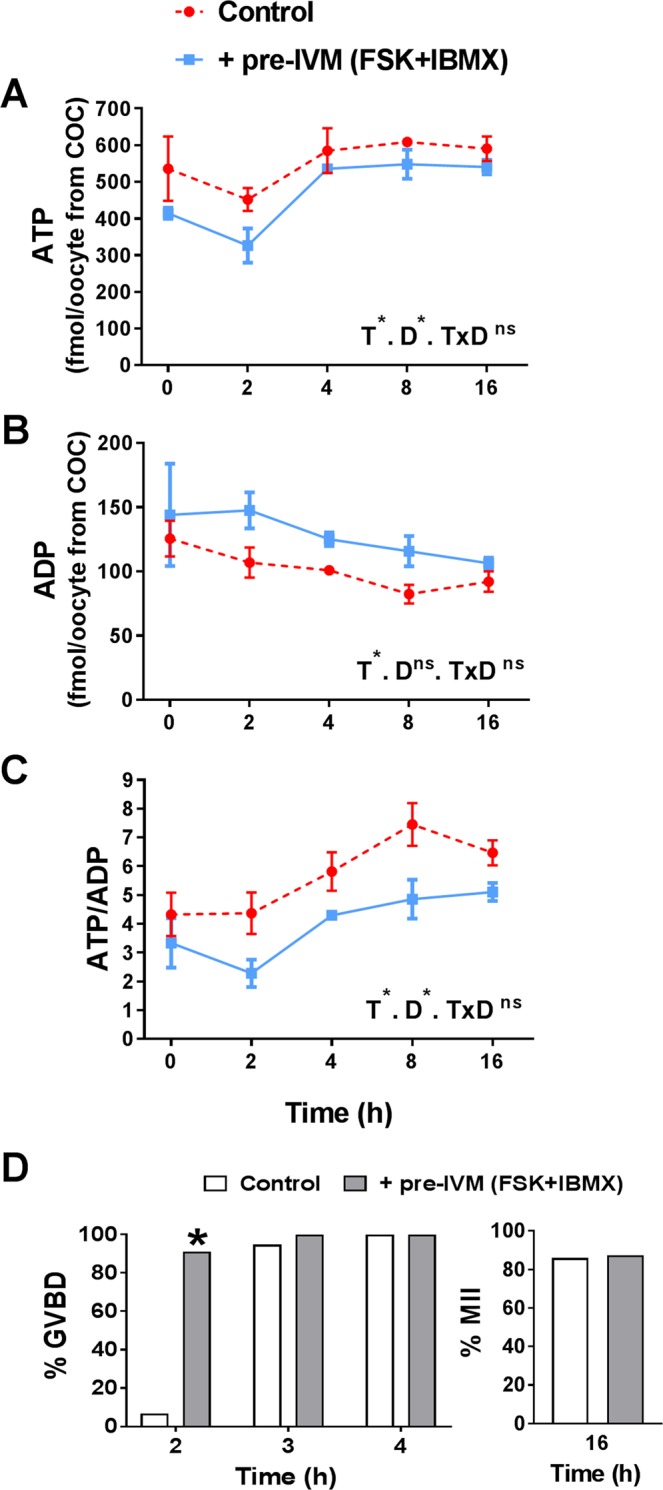
Elevated COC cAMP alters intra-oocyte adenine nucleotide levels throughout oocyte maturation. COCs were either not exposed (control, 0 h pre-IVM) or were exposed to 2 h of pre-IVM with FSK + IBMX prior to IVM culture in the presence of FSH for up to 16 h. Cumulus cells were then removed and intra-oocyte ATP (**A**), ADP (**B**), and the ATP:ADP ratio (**C**) were measured (mean ± SEM). T, treatment; D, oocyte culture duration; *Significantly different (P < 0.05, two-way ANOVA); ns, not significantly different. Oocyte meiotic maturation was assessed after 2, 3, 4 and 16 hours of culture (**D**). *Significantly different to control (P < 0.05, χ^2^). N = 3 biological replicates.

**Figure 4:**
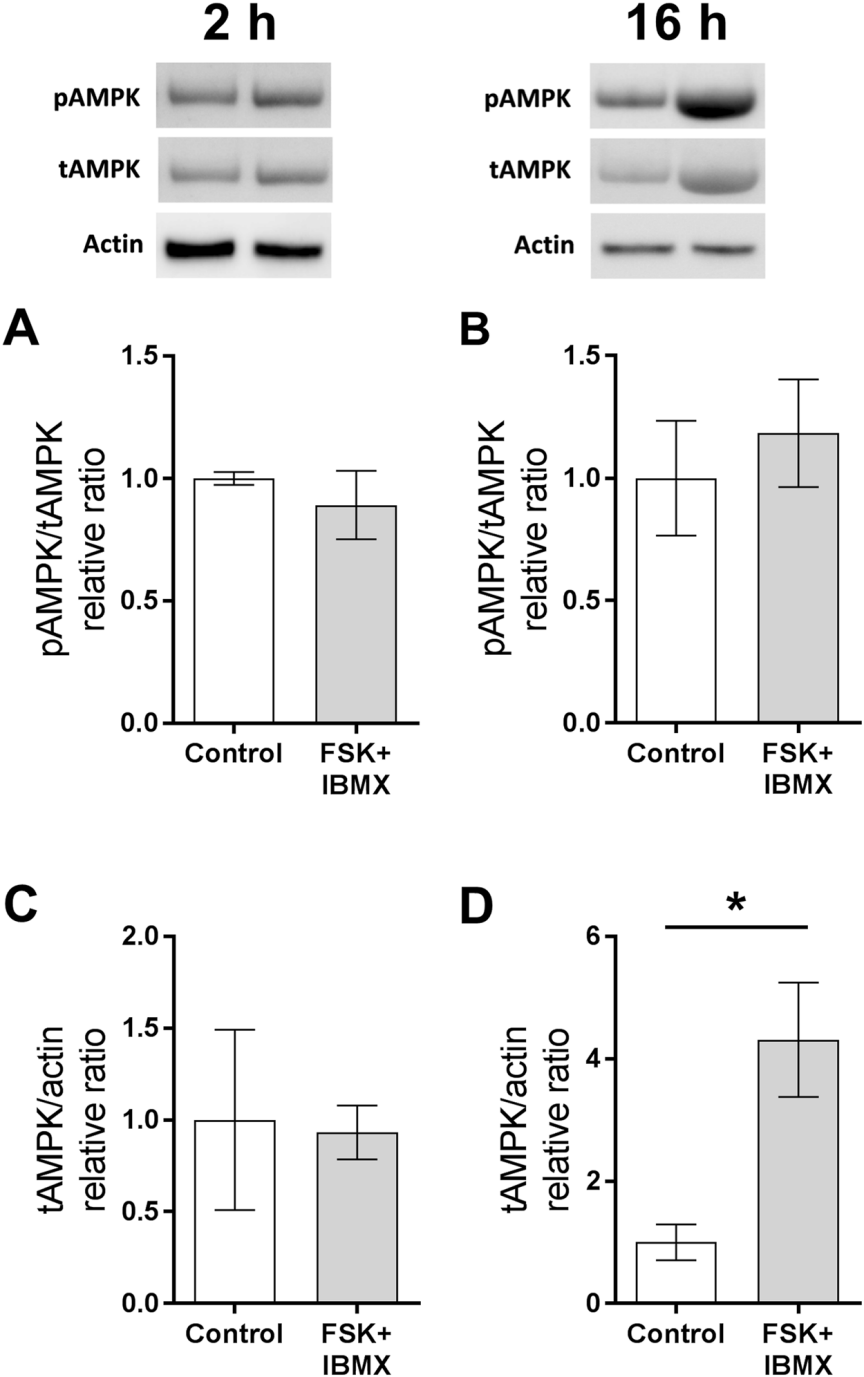
Elevated COC cAMP alters intra-oocyte AMPK production. COCs were either untreated (control) or pre-treated for 2 h (pre-IVM) with FSK + IBMX, then cultured without treatments in the presence of FSH for 2 h or 16 h. Cumulus cells were then removed and intra-oocyte phosphorylated (pAMPK) and total (tAMPK) AMPK were measured at 2 h (**A**,**C**) or 16 h (**B**,**D**). N = 3 biological replicates, data are mean ± SEM. *Significantly different (P < 0.05, t-test).

As treatment of COCs with FSK/IBMX (pre-IVM) leads to a large acute increase in cAMP^9,19^, it would be expected that adenine nucleotide substrates and metabolites would be affected in the oocyte. Oocyte adenine nucleotide and AMPK levels were measured ± pre-IVM to examine the impact of FSK/IBMX cAMP elevation. Oocytes from COCs (i.e. exposed to a FSK/IBMX treatment as intact COCs in the pre-IVM phase only followed by IVM, and then denuded of cumulus cells) exhibited significantly lower intra-oocyte ATP throughout oocyte maturation compared to controls (P < 0.05 main effect, two-way ANOVA; Fig. 3A). Oocyte ADP was significantly higher following FSK/IBMX pre-IVM than controls (P < 0.05 main effect, two-way ANOVA; Fig. 3B), and consequently the ATP:ADP ratio was significantly lower (P < 0.01 main effect, two-way ANOVA; Fig. 3C). Intra-oocyte ATP levels and the ATP:ADP ratio changed with duration of IVM (P < 0.005 main effects; Fig. 3A,C), associated with a drop in ATP at 2 hours of culture and a steady increase in the ATP:ADP ratio with time, regardless of treatment. Intra-oocyte AMP was below the limit of quantification, however, given that the ATP:ADP ratio is commonly used as a surrogate for ATP:AMP, one would anticipate that intra-oocyte AMP is also elevated in response to elevated COC cAMP. Oocytes remain meiotically arrested at the GV stage after pre-IVM with FSK/IBMX^16^, but then FSK/IBMX pre-IVM accelerated oocyte meiotic progression, with increased oocyte GVBD rates at 2 h of IVM relative to control (P < 0.01; Fig. 3D); GVBD rates were comparable by 3 h and MII rates at 16 h of culture. Oocytes exposed to FSK/IBMX pre-IVM exhibited significantly higher (P < 0.05) total AMPK at 16 h (Fig. 4D), but not at 2 h (Fig. 4C) of oocyte maturation. The ratio of phosphorylated AMPK (pAMPK) to total AMPK (tAMPK) was unaltered at both time points (Fig. 4A,B).

### Elevated COC cAMP increases COC ATP

**Figure 5:**
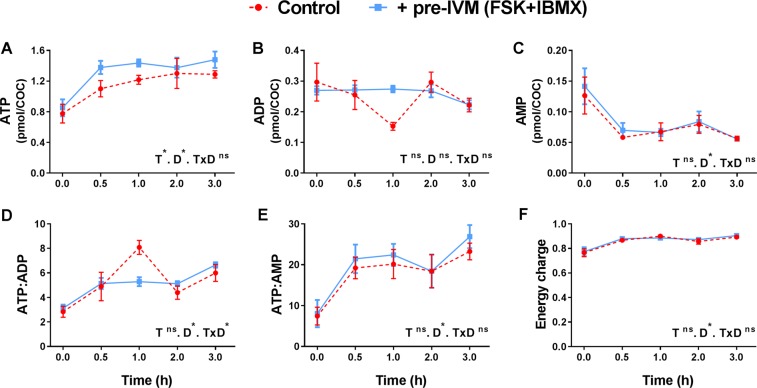
Elevated COC cAMP alters adenine nucleotide levels. COCs were either untreated (control) or pre-treated for 2 h (pre-IVM) with FSK + IBMX, then cultured without treatments in the presence of FSH for 3 h. COC ATP (**A**), ADP (**B**), AMP (**C**) were measured. COC ATP:ADP (**D**), ATP:AMP (**E**) ratios and energy charge (**F**) were calculated. N = 4 biological replicates, data are mean ± SEM. T, treatment; D, oocyte culture duration; *Significantly different (P < 0.05, two-way ANOVA); ns, not significantly different.

**Figure 6:**
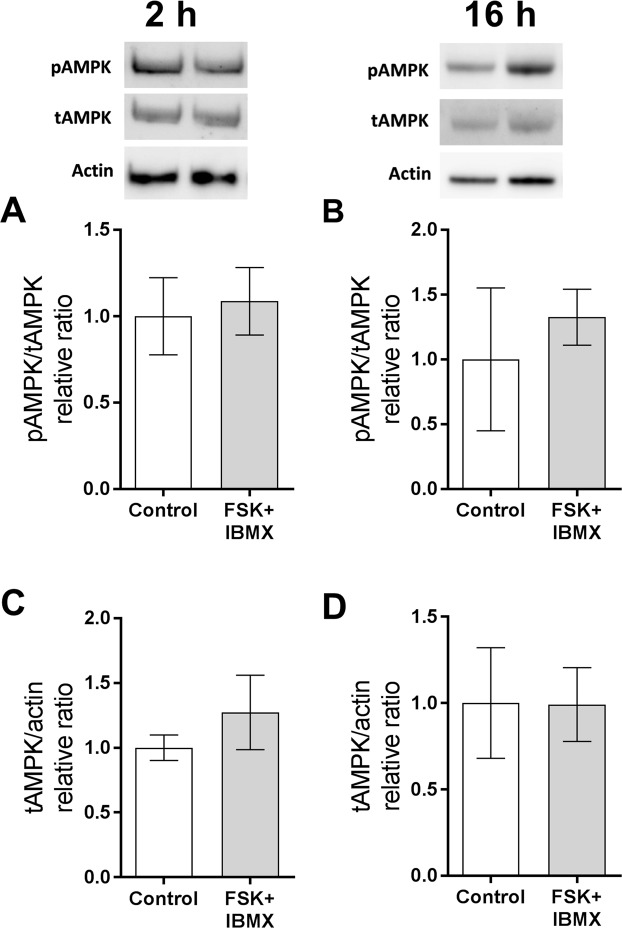
Elevated COC cAMP does not impact COC AMPK production and activity. COCs were either untreated (control) or pre-treated for 2 h (pre-IVM) with FSK + IBMX, then cultured without treatments in the presence of FSH for 2 h or 16 h. COC phosphorylated (pAMPK) and total (tAMPK) AMPK levels were measured at 2 h (**A**,**C**) or 16 h (**B**,**D**). N = 3 biological replicates, data are mean ± SEM. Data within graphs were not significantly different (P ˃0.05, t-test).

COC adenine nucleotide and AMPK levels were measured ± pre-IVM to examine the impact of FSK/IBMX cAMP elevation. In contrast to within the oocyte, ATP levels in the whole COC were significantly higher following FSK/IBMX pre-IVM treatment (P < 0.05 main effect, two-way ANOVA; Fig. 5A). There were no significant differences in ADP and AMP levels, or in the ATP:ADP and ATP:AMP ratios between control and FSK/IBMX pre-IVM treated COCs (Fig. 5B–E). There was also no significant difference in cellular energy charge between control and FSK/IBMX pre-IVM exposed COCs (Fig. 5F). Energy charge is an index of cellular energy status that is calculated by the relative levels of ATP, ADP and AMP^27^. Intra-oocyte energy charge was not obtainable since AMP was not quantifiable in denuded oocytes (Fig. 2). Irrespective of pre-IVM treatment, ATP and AMP levels changed over the first 3 hours of IVM, with COC ATP levels increasing and AMP levels decreasing, leading to time-dependent changes in the ATP:ADP and ATP:AMP ratios (all: P < 0.001 main effects; Fig. 5). Consistent with the lack of effect of FSK/IBMX pre-IVM on whole COC AMP, ADP, ATP/AMP or ATP/ADP, there was no significant difference in total or phosphorylated AMPK levels in control versus FSK/IBMX exposed COCs at 2 h or 16 h of oocyte maturation (Fig. 6). In summary, COC ATP was increased by FSK/IBMX pre-IVM, however no effect on AMPK was observed.

## Discussion

**Figure 7:**
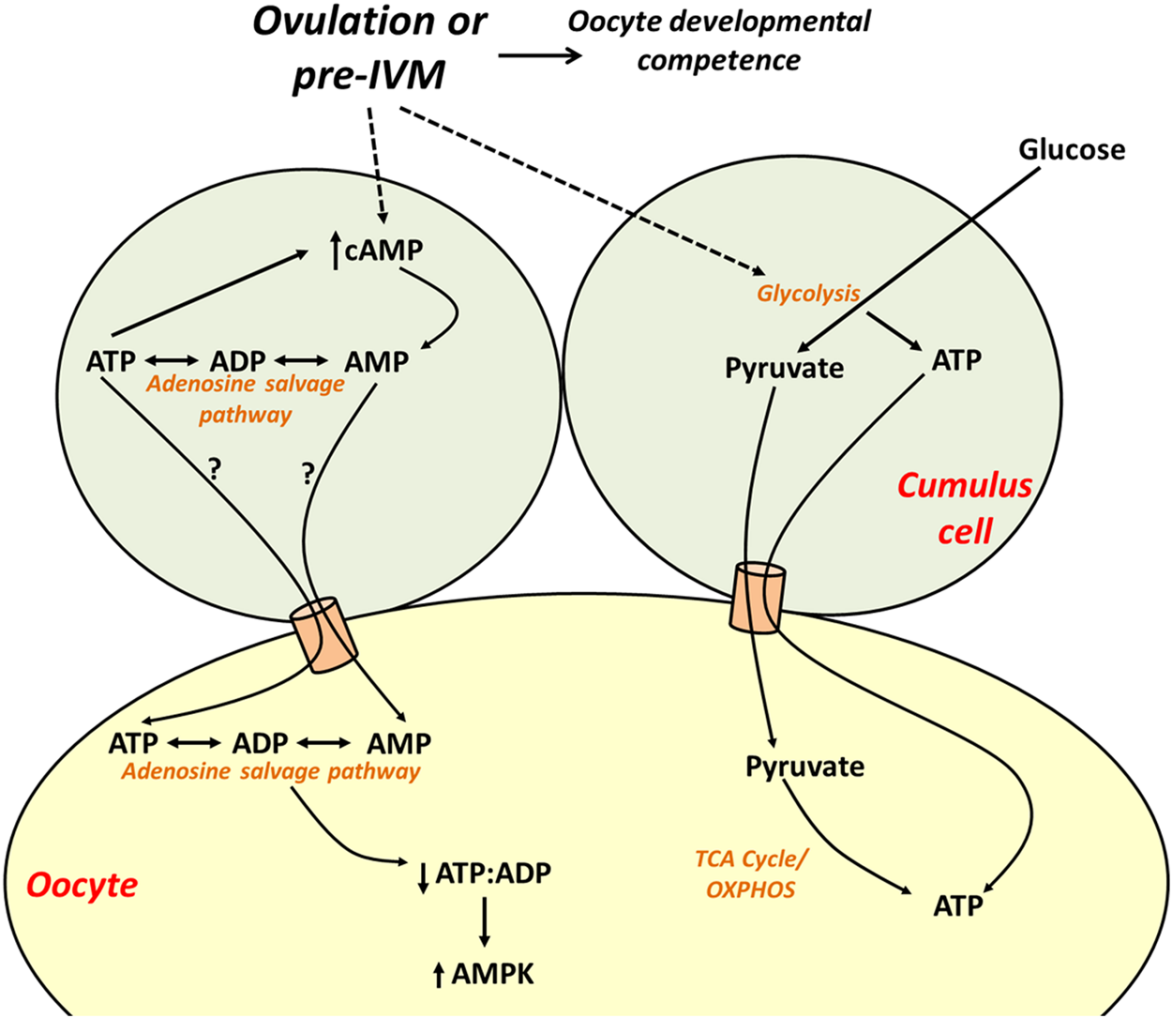
Hypothesised model of the impact of cAMP modulation in the cumulus-oocyte complex on oocyte energy production. Oocyte meiotic resumption and pharmacological cAMP upregulation via pre-IVM generate AMP from cAMP in cumulus cells which can be used via the adenosine salvage pathway to regenerate ATP in the oocyte. Upregulation of cAMP in the cumulus-oocyte complex leads to a decrease in intra-oocyte ATP, decreasing the ATP:ADP ratio and upregulating AMPK protein expression to restrain energy depletion. Pre-IVM also increases COC ATP by stimulating cumulus cell glycolysis. TCA, tricarboxylic acid cycle; OXPHOS, oxidative phosphorylation; AMPK, AMP-activated protein kinase.

Oocyte meiotic maturation is an energetically demanding process with large quantities of ATP required for metaphase I spindle formation and migration to the oocyte cortex^28^. Unlike cumulus cells, the oocyte has a very poor capacity for glycolysis and instead relies on cumulus cells to perform glycolysis and supply pyruvate as a substrate for oxidative phosphorylation by oocyte mitochondria for most metabolic energy (Fig. 7)^29^. Fully grown and maturing mammalian oocytes exhibit atypically immature mitochondria which are hooded, contain fewer cristae, and consequently have a reduced capacity for energy production via oxidative phosphorylation^30–32^. Ovulation and pre-IVM treatment with FSK/IBMX lead to marked increases in COC and intra-oocyte cAMP, with pre-IVM yielding ~4-fold higher COC cAMP than ovulatory LH^4,5,9^. Given that metabolism of intra-oocyte cAMP to AMP is required for meiotic resumption to occur (reviewed by Downs^2^), we postulated that there is potential for the oocyte to capitalise on the availability of large amounts of AMP following cAMP breakdown during oocyte meiotic maturation to generate ATP via the adenosine salvage pathway.

The adenosine salvage pathway, a two-step enzymatic process that phosphorylates AMP to ADP, and then ADP to ATP (Fig. 1), was recently proposed as an alternate pathway for ATP production in the maturing oocyte in order to meet its energy demands. Scantland, *et al*.^23^ recently provided evidence that bovine oocytes utilise this pathway for energy production, although some of the evidence provided was indirect as adenosine metabolite tracing was not performed^23^. In the current study we provided ^13^C_5_-AMP as a substrate for mouse COCs and denuded oocytes to directly trace its metabolism via the adenosine salvage pathway. Denuded oocytes and oocytes collected from intact COCs, cultured in the presence of ^13^C_5_-AMP, were found to contain ^13^C_5_-ATP and ^13^C_5_-ADP generated from this isotopic-labelled substrate. Furthermore, inhibition of creatine kinase (required for the ADP to ATP conversion step in the adenosine salvage pathway) completely ablated ^13^C_5_-ATP production without significantly affecting ^13^C_5_-ADP or ^13^C_5_-AMP levels. Hence these findings provide direct evidence to support the original hypothesis by Scantland, *et al*.^23^ that the adenosine salvage pathway is active in oocytes. However, while ^13^C_5_-ATP was generated from extracellular ^13^C_5_-AMP, the total amount of ATP in the cell did not change. This may be because the oocyte has fine control of ATP homeostasis, or utilisation of the exogenous AMP may be limited by the availability of other substrates in the adenosine salvage pathway. In support of the latter hypothesis, exogenous culture of denuded bovine oocytes with phosphocreatine, the phosphate donor in the adenosine salvage pathway, led to a significant increase in ATP levels^23^. Hence, taken together, it appears the adenosine salvage pathway is active in oocytes and it may be an additional means for the oocyte to generate ATP for energy demand, particularly during the energetically demanding process of meiotic maturation where a large amount of AMP is generated by phosphodiesterase breakdown of cAMP.

Cumulus cells play a major role in ATP production via the adenosine salvage pathway. The amount of ^13^C_5_-ATP was substantially higher in oocytes from COCs than in oocytes cultured without their cumulus vestment, and pharmacological uncoupling of gap junctions in the COC led to a 3.8-fold reduction in ^13^C_5_-ATP levels within the oocyte. These two observations suggest that cumulus cells likely directly supply the oocyte with ATP they generated via the adenosine salvage pathway (Fig. 7). In line with our observations, others have demonstrated that oocytes matured with intact cumulus cells have higher ATP than denuded oocytes^23,33^.

This study demonstrates that raising cAMP levels in COCs using forskolin and IBMX in a pre-IVM phase to maintain meiotic arrest induces energy deficit (decreased ATP and increased ADP/ATP ratio) within the oocyte. The lower intra-oocyte ATP concentrations immediately following cessation of FSK/IBMX treatment (0 h IVM) and the marked decrease during the early stages of meiotic resumption (0–2 h) can likely be attributed to the supraphysiological increased activity of adenylate cyclase by forskolin, which generates cAMP by using ATP as a substrate (Fig. 1). However, an additional factor contributing to ATP depletion may be a reduction in the amount of AMP available due to residual IBMX activity (blocking breakdown of cAMP to AMP), which would perturb ATP production from AMP via the adenosine salvage pathway. A sharp recovery in intra-oocyte ATP was noted between 2–4 h after pre-IVM which may be associated with IBMX clearance. Overall, using combined forskolin and IBMX may be a harsh approach to artificially inhibit oocyte meiotic resumption during pre-IVM. Furthermore, the success of FSK/IBMX pre-IVM in improving embryo development has proved variable in differing research settings^34^. Alternative approaches which do not activate adenylate cyclase are known to be effective in maintaining oocyte meiotic arrest in pre-IVM and lead to increased subsequent oocyte developmental competence. Culture with the synthetic cAMP analogue dibutyryl cAMP is effective in preventing germinal vesicle breakdown leading to enhanced oocyte quality^14^. More recently, a pre-IVM approach shown to be effective in preventing GVBD utilises the cGMP modulator c-type natriuretic peptide (CNP) as the meiosis arresting agent^35–37^. CNP is the natural follicular factor which maintains meiotic arrest in the antral follicle^38^. CNP-mediated pre-IVM has been shown to improve oocyte developmental competence in several species including human^35–37,39,40^, and likely represents the future of IVM since it more closely mimics the mechanisms of *in vivo* meiotic arrest and hence presents a more physiological approach than FSK/IBMX.

AMPK is a master energy sensing enzyme that directs metabolic changes in response to cellular energy status^24^. AMPK is activated by changes in the cellular ATP:AMP or ATP:ADP ratios and acts to restore energy balance by downregulating energy consuming anabolic pathways and upregulating energy producing catabolic pathways^24^. The increased energy demand (depletion in oocyte ATP, and consequent shift in ATP:ADP ratio) placed on the oocyte by generation of high levels of cAMP increased intra-oocyte AMPK synthesis, presumably by increasing mRNA translation during oocyte maturation, however the relative activity (phosphylation) of AMPK to total protein remained constant. The increase in total AMPK may be a means to restrain the ATP depletion caused by FSK exposure. In contrast to the oocyte, cAMP elevation in COC did not alter AMPK level, likely because ATP was elevated rather than depleted in the COC.

In contrast to intra-oocyte ATP, which decreased in response to pre-IVM, ATP levels in the whole COC were increased in response to elevated COC cAMP, demonstrating that cumulus cells respond differently to FSK/IBMX. Recent evidence suggests that elevating COC cAMP stimulates glucose uptake and metabolism in murine and bovine cumulus cells. FSK/IBMX pre-IVM leads to increased mouse COC lactate production throughout IVM, indicative of increased glycolysis^16,21^. In addition, microarray analysis of bovine cumulus cells following 6 h culture with FSK, IBMX and dipyridamole demonstrated a significantly altered gene expression profile relative to control, including the upregulation of genes involved in glucose uptake and metabolism (*GFPT2*, *HK2, SLC2A1*)^41^. Unlike cumulus cells, oocytes lack the glucose transporter SLC2A4 and hence have a poor capacity for glucose uptake and glycolysis^29^, and thus cannot capitalise on any stimulatory effects of high cAMP on these processes. Hence, elevated COC cAMP appears to induce metabolic alterations in cumulus cells which enhance energy production (despite consuming ATP as a substrate for cAMP synthesis), a phenomenon that has previously been associated with improved oocyte developmental outcomes^42–45^.

In conclusion, this study demonstrates that modulation of adenine nucleotides impacts mouse oocyte ATP production. Oocytes have the capacity to generate ATP from AMP via the adenosine salvage pathway, and cumulus cells enhance ATP production via this pathway. Furthermore, elevation of the AMP precursor cAMP impacts adenine nucleotide levels within the oocyte and cumulus cells, with these compartments responding differently. These findings contribute to our understanding of oocyte ATP metabolism and further elucidate the extent to which elevated cAMP, which occurs during ovulation and in using modern approaches to oocyte maturation *in vitro*, alters the metabolic properties of the COC. These new insights are important as they provide a new perspective in energy generation in the oocyte, which in turn is critical to oocyte developmental competence^29,46^, and are also significant for the development of new reproductive technologies such as oocyte IVM.

## Materials and Methods

### Cumulus-oocyte complex collection

Mice were maintained in accordance with the Australian Code of Practice for Care and Use of Animals for Scientific Purposes and all experimental protocols were approved by the University of New South Wales Animal Care & Ethics Committee. 129/Sv mice were used for pre-IVM experiments and C57/Bl6 mice were used for metabolite tracing experiments. Peri-pubertal 28–30-day old females were given an intraperitoneal injection of 5 IU of equine chorionic gonadotropin (Folligon, Intervet, Boxmeer, The Netherlands). Ovaries were collected in HEPES-buffered alpha minimum essential medium (αMEM; Gibco, Life Technologies, New York, USA) supplemented with 3 mg/mL BSA 46 h post-eCG. COCs were released from preovulatory follicles using a 27-gauge needle into HEPES-buffered αMEM (Gibco) with 3 mg/mL of BSA and 100 µM of IBMX (Sigma-Aldrich, Merck, Darmstadt, Germany), and collected using flame-pulled borosilicate Pasteur pipettes.

### COC culture and pre-IVM treatments

In experiments examining the consequences of elevated COC cAMP, COCs either underwent standard IVM (control, no pre-IVM) or were pre-treated for 2 h with forskolin and IBMX (pre-IVM) prior to IVM. Control (no pre-IVM) COCs were freshly collected from follicles, washed three times and immediately placed into IVM culture drops (bicarbonate-buffered αMEM containing 3 mg/mL BSA and 50 mIU/mL FSH (Puregon, Organon, The Netherlands)). Control (no pre-IVM) COCs were not exposed to any pre-IVM duration, and were therefore placed into IVM culture 2 h before the pre-IVM group. For the pre-IVM group, COCs were collected from follicles, then cultured in the presence of 50 µM FSK (Sigma-Aldrich) and 50 µM IBMX (Sigma-Aldrich) for 2 h at 37 °C in 5% CO_2_ in air before washing and transfer into IVM media (bicarbonate-buffered αMEM containing 3 mg/mL BSA and 50 mIU/mL FSH) for IVM culture. Pre-IVM treatment is an established research and clinical protocol used to generate mature oocytes *in vitro* for IVF and embryo transfer^9,20–22,47^. The doses of FSK and IBMX used were based on previous studies^9,21^. Following ± pre-IVM, all COCs underwent IVM by culturing at a volume of ~10 µL/COC in bicarbonate-buffered αMEM (Gibco) supplemented with 3 mg/mL BSA and 50 mIU/mL FSH (Puregon, Organon, The Netherlands). Following IVM ± pre-IVM, groups of intact COCs were either analysed whole or the oocytes were denuded of cumulus cells (oocyte from COC) and analysed.

### Culture of COCs and denuded oocytes with ^13^C_5_-AMP

In experiments examining conversion of AMP to ATP, COCs or denuded oocytes were cultured for 3 h in bicarbonate-buffered αMEM (Gibco) supplemented with 3 mg/mL BSA, 50 mIU/mL FSH ± ¹³C_5_-adenosine 5′-monophosphate (^13^C_5_-AMP; cat# A281782; Toronto Research Chemicals, Canada) ± 2 mM iodoacetamide (IAC; Sigma) or 200 µM carbenoxolone (CBX; Sigma). A concentration of 1 mM ¹³C_5_-AMP was used for all experiments except the dose response experiment where doses are indicated. The concentrations of ^13^C_5_-AMP and IAC were based on previous studies^23,48^. Following culture, denuded oocytes, or oocytes from COCs were collected and adenine nucleotides were extracted as described below.

### Adenine nucleotide extraction and quantification by LC-MS/MS

ATP, ADP, and AMP were extracted from cells, processed and quantified by liquid chromatography with tandem mass spectrometry detection (LC-MS/MS), as we recently described^49^. Briefly, following culture, whole COCs or oocytes were placed into ice-cold 80% methanol, and sonicated on ice for 1 min. Samples were then incubated at −20 °C for 20 min and centrifuged at 14,000 g for 10 min at 4 °C. Supernatants were collected, stored at −80 °C, and evaporated to dryness using a Savant SpeedVac vacuum centrifuge. Samples were spiked with 20 µL of 400 nM isotope-labelled internal standards (^13^C_5_-AMP, ^15^N_5_-ADP and ^13^C_10_^15^N_5_-ATP) prior to drying. Samples were then reconstituted in 1 mL of 100 mM ammonium acetate and loaded on to a solid phase extraction column (Hypersep Hypercarb 50 mg/1 mL, Thermo Scientific) preconditioned with 1 mL of 60% v/v acetonitrile in formic acid buffer (0.3% v/v formic acid adjusted to pH 9 with ammonia), then with 1 mL of ultrapure water. The cartridge membrane was washed with 1 mL water, then with 300 µl of 60% acetonitrile. Analytes were eluted in 500 µL of 60% acetonitrile in formic acid buffer (0.3% formic acid at pH 9) and evaporated to dryness. Dried samples were reconstituted in 50–100 µl of 100 mM ammonium acetate. Analytes were separated and quantified by LC-HESI-MS/MS as previously described^49^.

The uptake of ^13^C_5_-AMP and its conversion to ¹³C_5_ adenosine 5′-diphosphate (^13^C_5_-ADP) and ¹³C_5_ adenosine 5′-triphosphate (^13^C_5_-ATP), as well as endogenous ATP, were determined by targeting the adenine moiety (*m/z 136.2*) generated in positive ion mode heated electrospray ionisation (HESI) product scan. ^13^C_10_, ^15^N_5_ 5′-guanosine monophosphate (Sigma-Aldrich) was used as an internal standard for the quantitation of endogenous and ¹³C_5_ labelled nucleotides. Table 1 shows monitored transitions and collision energy used.

**Table 1:** LC-MS/MS monitored transitions (*m/z*) and collision energy used for stable isotope tracing.

Nucleotide	(*m/z*)	Collision Energy (V)
^13^C_5_-AMP	353 > 136.2	25
^13^C_5_-ADP	433 > 136.2	25
^13^C_5_-ATP	513 > 136.2	25
ATP	508.2 > 136.2	25
^13^C_10_,^15^N_5_ 5′-GMP	379 > 162.0	20

### Measurement of total and phosphorylated αAMPK

Following 2 h or 16 h of COC IVM ± pre-IVM, whole COCs or oocytes were lysed in a protein lysis buffer (10 mM Tris, 150 mM NaCl, 1 mM EDTA, 1% Triton X-100) containing complete protease inhibitor (Roche) and 10 μl/mL of phosphatase inhibitor (Sigma-Aldrich) cocktails. Samples were mixed with loading buffer containing 100 mM dithiothreitol, freeze-thawed 4 times on dry ice and boiled at 95 °C for 5 min. Proteins were separated by electrophoresis using an SDS PAGE Bolt gel (Thermo Fisher Scientific, Waltham, MA), then transferred onto polyvinylidene fluoride membranes (Merck Millipore, Darmstadt, Germany) and blocked for 1 h using tris-buffered saline buffer with 0.05% Tween-20 (TBST) and 3 mg/mL of BSA. They were then incubated over night at 4 °C with anti-phospho-AMPKα (Thr172) rabbit mAb that detects α1 and α2 isoforms (cat# 2535 S; Cell Signalling Technology, Danvers, USA) at 1:1000 dilution in blocking solution. After washing in TBST, membranes were incubated at room temperature for 4 hours in goat anti-rabbit HRP-conjugated secondary antibody (Santa Cruz Biotechnology, Dallas, USA) diluted to 1:3000. The bands were detected using Amersham ECL Prime (GE healthcare, UK) and imaged using the ImageQuantTM Las4000 (GE Healthcare, UK). Membranes were then incubated with a stripping buffer (200 mM glycine, 3.5 mM SDS, 1% Tween20, pH 2.2), blocked and incubated overnight with rabbit anti-AMPKα1 (cat# 07-350; Merck Millipore) at 1:1000 dilution followed by incubation using goat anti-rabbit HRP-conjugated secondary antibody (Santa Cruz Biotechnology). The membrane was then stripped, blocked, and incubated overnight with mouse anti-actin antibody (cat# MAB1501R; Merck Millipore) at 1:1000 dilution and incubated with a donkey anti-mouse HRP-conjugated secondary antibody (Santa Cruz Biotechnology) at a 1:10,000 dilution. Relative band intensity was measured using ImageJ software (National Institute of Health, USA). Total AMPK band intensities were normalised to actin band intensities, whilst phosphor-AMPK band intensities were normalised to total AMPK band intensities. Results are represented as the mean ± SEM of 3 replicate experiments.

### Assessment of meiotic stage

At specified time points, oocytes were denuded of cumulus cells by mechanical shearing using a P200 pipette. Nuclear maturation was scored using an inverted microscope as germinal vesicle stage (GV), germinal vesicle breakdown (GVBD; when the GV is not visible), MII (where the first polar body is observed in the perivitelline space), or degenerated.

### Statistical analyses and calculations

Statistical analyses were conducted using GraphPad Prism 7 software. For experiments where two treatments were compared, differences were analysed by a t-test. Where two independent variables were tested, a two-way ANOVA was used. All data are presented as the mean ± SEM. For meiotic stage assessment, statistical significance was assessed using χ2 testing. Probabilities of P < 0.05 were considered significant. COC cellular energy charge was calculated using the following equation^27^:

$${\rm{Energy}}\,{\rm{charge}}=\frac{[{\rm{ATP}}]+\frac{1}{2}[{\rm{ADP}}]}{[{\rm{ATP}}]+[{\rm{ADP}}]+[{\rm{AMP}}]}$$

### Summary statement

Oocyte meiotic resumption generates AMP from cAMP which the oocyte can use to regenerate ATP via the adenosine salvage pathway. Cumulus cells enhance this process.

## Supplementary information

Supplementary Information:Western blots

## Data Availability

The datasets generated during and/or analysed during the current study are available from the corresponding author on reasonable request.
