# Supplementary material for: Participation of the adenosine salvage pathway and cyclic AMP modulation in oocyte energy metabolism
Source: Sci Rep. 2019 Dec 5;9:18395. doi: 10.1038/s41598-019-54693-y (PMC6895058; doi:10.1038/s41598-019-54693-y)

Participation of the adenosine salvage pathway and cyclic AMP modulation in oocyte energy metabolism

Dulama Richani[1*], Cathy F. Lavea[1], Raji Kanakkaparambil[1,2], Angelique H. Riepsamen[1], Michael J. Bertoldo[1,3], Sonia Bustamante[4], Robert B. Gilchrist[1]

Supplemental information: Full l length Western blot images.

Actin COCs 16h

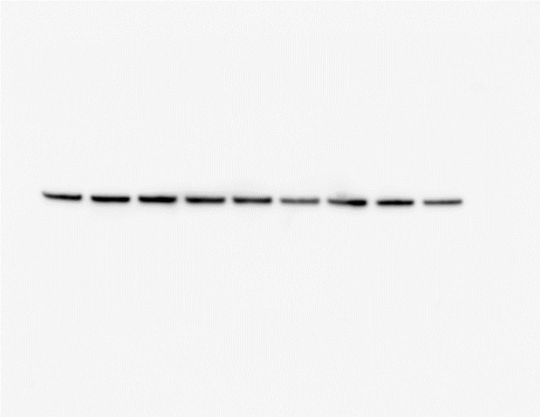

Actin CDOs 16h

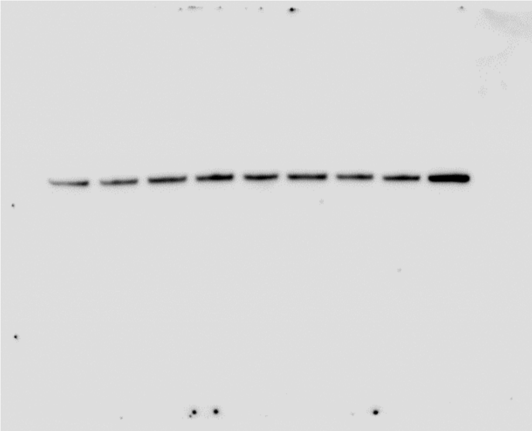

tAMPK COCs 16h

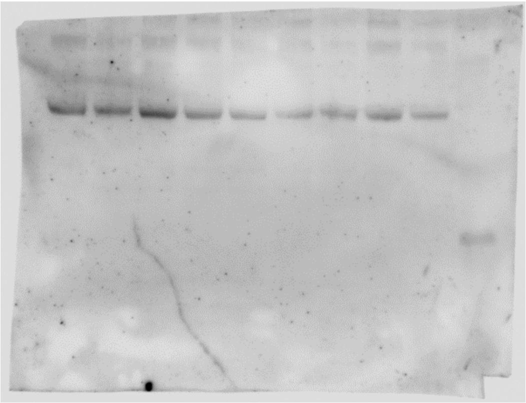

tAMPK CDOs 16h

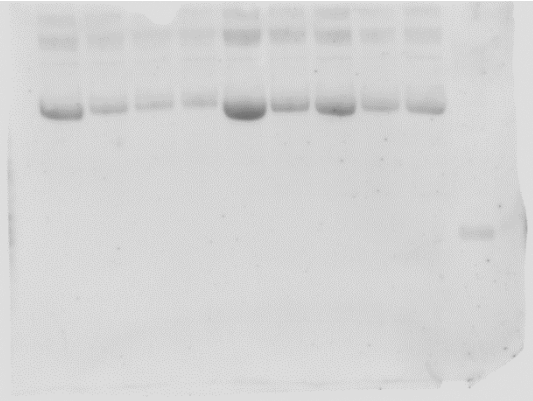

pAMPK COCs 16h

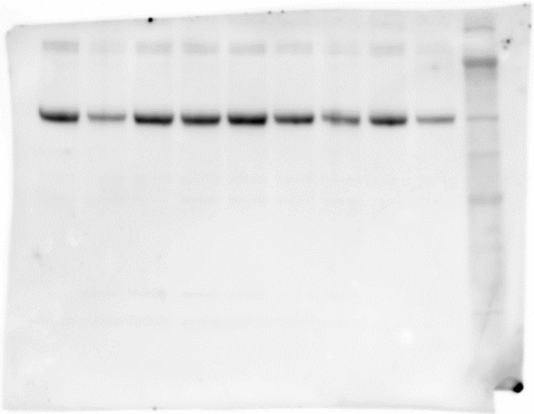

pAMPK CDOs 16h

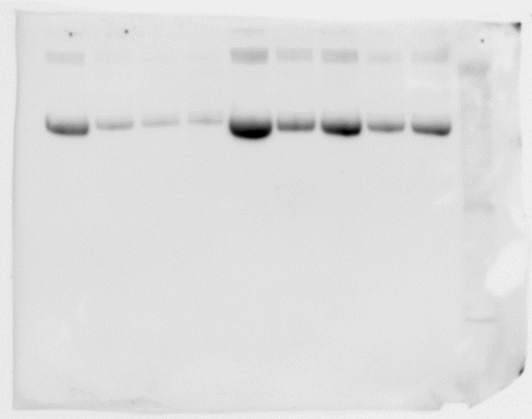

Actin COC 2h

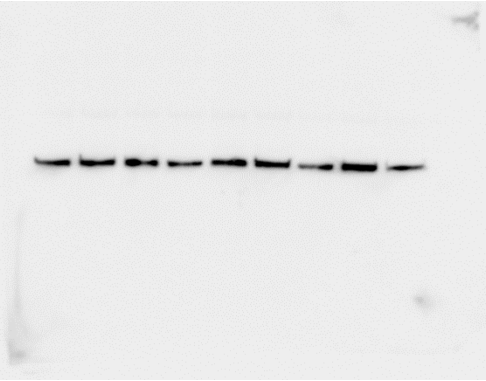

Actin CDOs 2h

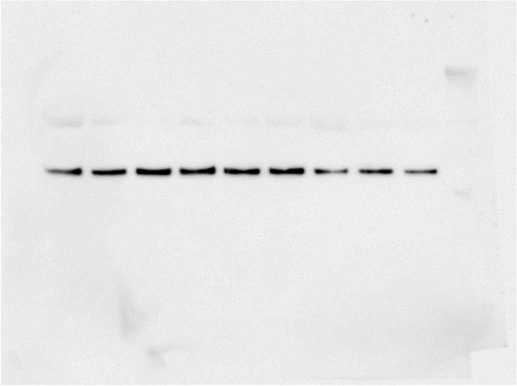

tAMPK COCs 2h

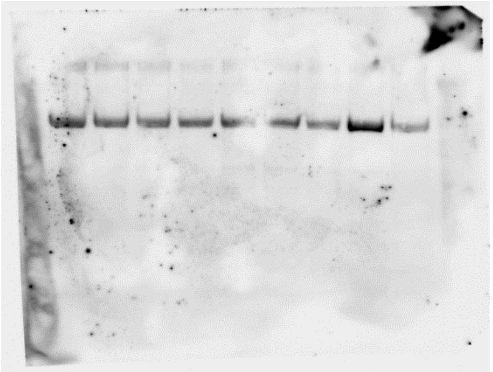

tAMPK CDOs 2h

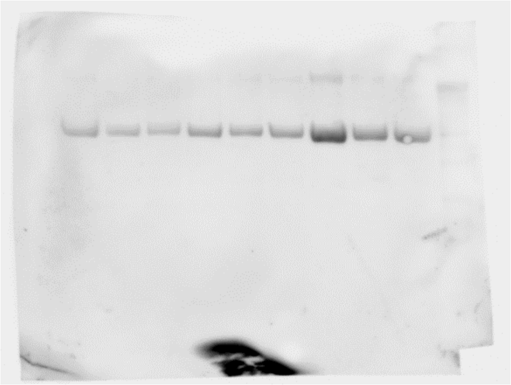

pAMPK COCs 2h

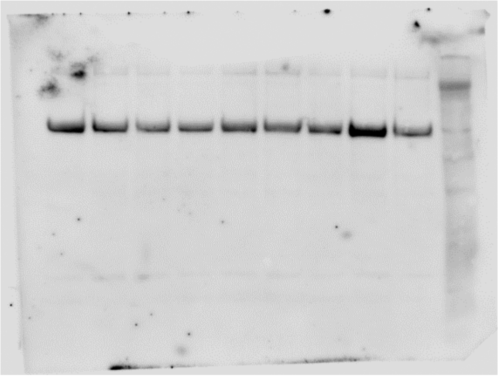

pAMPK CDOs 2h

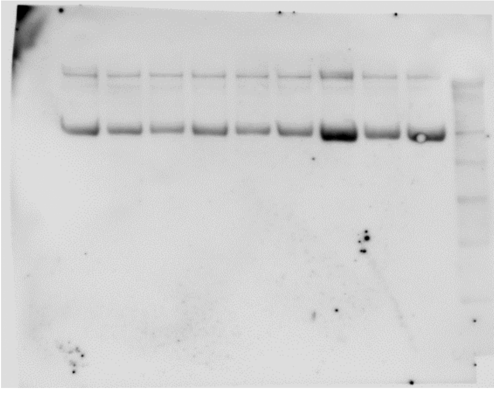

Supplement: Supplementary file 1 — Supplementary Information:Western blots [file 41598_2019_54693_MOESM1_ESM.pdf]
